# Cross design analysis of randomized and observational data – application to continuation rates for a contraceptive intra uterine device containing Levonorgestrel in adolescents and adults

**DOI:** 10.1186/s12905-018-0674-1

**Published:** 2018-11-09

**Authors:** Tatsiana Vaitsiakhovich, Anna Filonenko, Richard Lynen, Jan Endrikat, Christoph Gerlinger

**Affiliations:** 1 RWE Strategy & Outcomes Data Generation, Bayer AG, Müllerstraße 178, 13353 Berlin, Germany; 2 Market Access Pulmonology and Women’s Healthcare, Bayer AG, Müllerstraße 178, 13353 Berlin, Germany; 3 US Medical Affairs Women’s Healthcare, Bayer U.S. LLC, 100 Bayer Boulevard, Whippany, NJ 07981 USA; 4 Radiology, Bayer AG, Müllerstraße 178, 13353 Berlin, Germany; 5 Gynecology, Obstetrics and Reproductive Medicine, University of Saarland Medical School, 66421 Homburg, Saar Germany; 6 Statistics and Data Insights, Bayer AG, Müllerstraße 178, 13353 Berlin, Germany

**Keywords:** Cross design syntheses, Continuation rates, Intra-uterine device

## Abstract

**Background:**

To combine results from a randomized controlled study (RCT) and an observational study (OS) to evaluate discontinuation rate of a levonorgestrel-containing intrauterine contraceptive device (LNG IUD) in a real-life setting.

**Methods:**

We included 253 parous and nulliparous women aged 21–40 years from our own phase II RCT. A total of 1607 women of all ages (including adolescents, < 20 years) were recruited from an OS. We applied the cross design synthesis (CDS) method recommended by the United States General Accounting Office. This method combines the different strengths of RCTs and OSs into one single estimate.

**Results:**

Combined continuation rates for parous vs nulliparous women could be estimated more precisely as well as overall continuation rates after one (86.6%) and two years (78.5%), irrespective of age and parity.

**Conclusion:**

Cross design synthesis allowed more precise estimation of continuation rates of an intrauterine device.

## Background

For assessing the risk benefit profile of medical technologies, today, multiple data sources are leveraged, e.g., randomized clinical trials (RCT), observational studies (OS), health insurers’ claims data, etc. Data from these different sources are usually analyzed separately.

RCTs, mostly performed as phase II or phase III studies in the framework of clinical development programs, typically recruit a highly selected subset of the total patient population in order to achieve the highest possible internal validity. However, as a consequence, the external validity is low. Therefore, findings in patients purposely excluded from RCTs should complement the body of data. These patients are usually included in OSs, which are more representative of the daily routine clinical setting. Thus, OSs focus on external validity while their internal validity is compromised by confounding factors which can hardly be controlled.

There is uncertainty whether differing treatment effects observed in RCTs and OSs are due to different inclusion criteria or are caused by other hard to specify ‘real life’ factors. In the pursuit of raising internal and external validity of parameters like treatment efficacy, overall safety, treatment adherence, quality of life, etc. for informed healthcare decisions, combining data from RCTs and OSs is an avenue to go.

Research on different methods for merging data from RCTs and from OSs into one single analysis experienced increasing attention in the biomedical and biostatistical literature [1–3]. One of these methods is has been coined ‘cross design synthesis’ (CDS).

The aim of our study was to apply the method of CDS on investigating continuation rates of the levonorgestrel-containing intrauterine device (LNG IUD) (Mirena®), indicated for long-term reversible contraception. This LNG IUD provides higher contraceptive effectiveness than typical oral contraceptives as it does not rely on regular user adherence which is of special relevance for adolescents. As adherence and continuation are key for effectiveness [4] we investigated one and two year continuation rates of this LNG IUD by merging results from an RCT and an OS.

## Methods

For our CDS we leveraged results from our own RCT and from an OS, published by Abraham et al. [5]. To our knowledge, these were the only large scale data sources available for our research question.

The RCT was a randomized clinical phase II study comparing 3 doses of the LNG IUD in a parallel open-label design. A total of 733 parous and nulliparous women, aged 21–40 years, seeking long term contraception, were included. Of those, 253 women took the LNG IUD analyzed in this study. Follow-up was for three years. The primary target parameter was contraceptive efficacy, measured by the Pearl Index [6]. The main results of the study were published by Gemzell-Danielsson et al. [7].

The observational data were taken from the Contraceptive CHOICE Project - a prospective cohort study that followed 9256 participants with telephone surveys at the Washington University School of Medicine in St. Louis. At enrollment women at risk of unintended pregnancy could start a new reversible contraceptive method after comprehensive counselling on all available reversible contraceptive options. Details of the Contraceptive CHOICE study have been published elsewhere [8]. We used the subset of continuation rates of the LNG IUD as data from the real-world setting for our CDS (Table 1). The individual patient data for the RCT were re-analyzed retrospectively to match the definitions used in the analysis of the OS as described in [5, 8].

**Table 1:** Percentage of women continuing levonorgestrel IUD at 1 and 2 years by age and parity in the OS (modified from Abraham et al. 2015, [5])

Age group and parity	n	Continuation rate year 1 (in %)	Continuation rate year 1 (95%-Confidence Interval)	Continuation rate year 2 (in %)	Continuation rate year 2 (95%-Confidence Interval)
> 25 parous	1271	86	(84; 87)	76	(74; 78)
> 25 nulliparous	395	87	(83; 90)	77	(72; 81)
20–25 parous	851	86	(83; 88)	73	(73; 76)
20–25 nulliparous	850	87	(85; 89)	79	(79; 82)
< 20 parous	121	82	(73; 88)	73	(63; 80)
< 20 nulliparous	241	81	(76; 86)	67	(61; 73)

CDS was initially recommended by the United States General Accounting Office as a new strategy for medical effectiveness research [9, 10]. The aim is to combine the different strengths of RCTs and OSs into one single estimate by ‘extrapolating’ the RCT results to the initially excluded population by using OS data.

Simplifying the approach of Kaizar [3] we calculated the cross design estimator based on the results from the RCT *d*_*RCT*_ and the results from the OS *d*_*OS*_ stratified according to whether or not the women of the OS fulfilled the RCT’s major inclusion criterion, i.e. age of at least 20. The results for these strata were denominated as ${d}_{OS_{in}}$ for the women of the OS fulfilling the RCT’s inclusion criterion and as ${d}_{OS_{ex}}$ for the women of the OS not fulfilling the RCT’s inclusion criterion. The CDS estimator can be written as

$$ {d}_{CDS}={d}_{RCT}+\frac{n_{OS_{ex}}}{n_{OS}}\times \left({d}_{OS_{ex}}-{d}_{OS_{in}}\right) $$

where *n*_*OS*_ stands for the number of women included in the OS and ${n}_{OS_{ex}}$ for the number of women that were included in the OS and that did not fulfil the RCT’s inclusion criterion. The variance ${S}_{CDS}^2$ of the CDS estimator can be expressed as

$$ {S}_{CDS}^2=\frac{S_{RCT}^2}{n_{RCT}}+\frac{n_{OS_{ex}}^2}{n_{OS}^2}\times \left(\frac{S_{OS_{ex}}^2}{n_{OS_{ex}}}+\frac{S_{OS_{in}}^2}{n_{OS_{in}}}\right) $$

where ${S}_s^2$ and *n*_*s*_ stand for the variance and sample size of the women belonging to the subscripted group *s*.

The CDS estimator is unbiased if treatment selection error for the patients in the OS, fulfilling the inclusion criteria of the RCT, and the patients, who do not, is constant [3]. The CDS continuation rates after one and two years of LNG IUD use were calculated for parous and nulliparous women as well as for the total population.

## Results

Table 1 shows the results from the observational study on the LNG IUD published by Abraham et al. [5]. We selected continuation rates after 1 and 2 years grouped by age and parity as basis for our calculation.

As a first step we condensed the findings of the OS in two age groups: > 20 years (i.e. those and 20–25 and > 25 years) and < 20 years and added the respective results from the RCT (Table 2). The sample size of the OS was much higher (*n* = 1607) than for the RCT (*n* = 253).

**Table 2:** Pooled (age groups 20–25 and > 25) continuation rates (%) from the OS and RCT by age and parity

Data source	Age group and parity	n	Continuation rate year 1 (in %)	Continuation rate year 1 (95%-Confidence Interval)	Continuation rate year 2 (in %)	Continuation rate year 2 (95%-Confidence Interval)
OS	> 20 parous	2122	86	(84.65; 87.34)	74.79	(73.45; 76.13)
	> 20 nulliparous	1245	87	(85.24; 88.75)	78.36	(76.60; 80.12)
	< 20 parous	121	82	(74.50; 89.50)	73.00	(64.50; 81.50)
	< 20 nulliparous	241	81	(76.00; 86.00)	67.00	(61.00; 73.00)
		3729				
RCT	> 20 parous	203	90.6	(86.58; 94.61)	81.49	(76.10; 86.88)
	> 20 nulliparous	50	74.0	(61.84; 86.15)	70.00	(57.29; 82.70)
	< 20 parous	0	NA	NA	NA	NA
	< 20 nulliparous	0	NA	NA	NA	NA
		253				

First, women > 20 years and parity: In the OS nulliparous women showed slightly higher continuation rates than parous women, especially visible after two years, 78% vs. 75%. In the RCT this trend was the other way round: parous women showed higher continuation rates than nulliparous women, i.e. 81% vs 70% after two years.

Comparing study types, parous women showed higher continuation rates in the RCT than in the OS, while it was the other way round for nulliparous women.

Secondly, women < 20 years and parity (only OS results available): Nulliparous women had lower continuation rates than parous women, e.g. 67% vs 73% after two years. The continuation rates of the younger women were consistently lower than for those 20 years and older.

Irrespective of parity the continuation rates for women > 20 years were lower in the OS than in the RCT, e.g., 76% vs 79% after 2 years.

In the OS the continuation rates for women < 20 years of age were lower than those for women > 20 years, 69% vs 76% after two years (Table 3).

**Table 3:** Pooled (age groups 20–25 and > 25) continuation rates (%) from OS and RCT by age

Data source	Age group	n	Continuation rate year 1 (in %)	Continuation rate year 1 (95%-Confidence Interval)	Continuation rate year 2 (in %)	Continuation rate year 2 (95%-Confidence Interval)
OS	> 20	3367	86.36	(85.30, 87.43)	76.11	(75.05, 77.18)
	< 20	362	81.33	(77.16, 85.50)	69.00	(64.10, 73.90)
RCT	> 20	253	87.32	(83.20, 91.43)	79.22	(74.18, 84.25)
	< 20	NA	NA	NA	NA	NA

The cross design synthesis combines results of the two study types by parity but not by age, as women < 20 years were not included in the RCT.

The CDS continuation rates for parous women were 90 and 81% after one and two years, respectively. The corresponding figures for nulliparous women were lower, i.e., 73 and 68%. For all women, irrespective of parity, the CDS continuation rates were 87% after the first year and 79% after the second year (Table 4).

**Table 4:** Results cross design synthesis, continuation rates (%) by parity

Parity	Continuation rate year 1 (in %)	Continuation rate year 1 (95%-Confidence Interval)	Continuation rate year 2 (in %)	Continuation rate year 2 (95%-Confidence Interval)
Parous	90.38	90.07; 90.69	81.39	80.98; 81.80
Nulliparous	73.02	72.20; 73.85	68.15	67.29; 69.01
Combined	86.83	86.57; 87.09	78.52	78.21; 78.84

## Discussion

Merging of data from RCTs and OSs offers new possibilities to assess medical interventions in a broader sense. CDS combines results from studies with complementary designs in order to capture the designs’ strengths and minimize the studies’ weaknesses. We used this method to estimate continuation rates of an LNG IUS. To the best of our knowledge, this is the first study of this kind on any long-acting contraceptive.

We were able to enrich the RCT’s results for women > 20 years with data for women of all age groups, including adolescents (< 20 years), from an OS. Numerically the results did not change drastically but this was probably due to the low fraction of adolescents in the OS (9.7%). However, we obtained a more precise estimate of the continuation rates.

A limitation of our study is that only our RCT provided individual data sets while we could only use aggregated data from the OS. Therefore, only matching of the study populations for age was possible. Other comparisons were impossible as the study populations differed substantially, e.g. for ethnic origin: The RCT population was almost exclusively Caucasian whereas 48.1% of the OS population was Black [5]. Also for geographic location: The RCT was performed in northern and central Europe while the OS was performed in St. Louis, Missouri, USA.

## Conclusion

Cross design synthesis allowed more precise estimation of continuation rates of an intrauterine device.

## Abbreviations

CDS: Cross design synthesis
LNG IUS: Levonorgestrel-containing intrauterine contraceptive device
OS: Observational study
RCT: Randomized controlled study
